# Respiratory Complex I in *Bos taurus* and *Paracoccus denitrificans* Pumps Four Protons across the Membrane for Every NADH Oxidized*

**DOI:** 10.1074/jbc.M116.771899

**Published:** 2017-02-07

**Authors:** Andrew J. Y. Jones, James N. Blaza, Febin Varghese, Judy Hirst

**Affiliations:** From the Medical Research Council Mitochondrial Biology Unit, Cambridge, CB2 0XY, United Kingdom

**Keywords:** complex I, electron transfer complex, mitochondria, mitochondrial respiratory chain complex, proton motive force, oxidative phosphorylation

## Abstract

Respiratory complex I couples electron transfer between NADH and ubiquinone to proton translocation across an energy-transducing membrane to support the proton-motive force that drives ATP synthesis. The proton-pumping stoichiometry of complex I (*i.e.* the number of protons pumped for each two electrons transferred) underpins all mechanistic proposals. However, it remains controversial and has not been determined for any of the bacterial enzymes that are exploited as model systems for the mammalian enzyme. Here, we describe a simple method for determining the proton-pumping stoichiometry of complex I in inverted membrane vesicles under steady-state ADP-phosphorylating conditions. Our method exploits the rate of ATP synthesis, driven by oxidation of NADH or succinate with different sections of the respiratory chain engaged in catalysis as a proxy for the rate of proton translocation and determines the stoichiometry of complex I by reference to the known stoichiometries of complexes III and IV. Using vesicles prepared from mammalian mitochondria (from *Bos taurus*) and from the bacterium *Paracoccus denitrificans*, we show that four protons are pumped for every two electrons transferred in both cases. By confirming the four-proton stoichiometry for mammalian complex I and, for the first time, demonstrating the same value for a bacterial complex, we establish the utility of *P. denitrificans* complex I as a model system for the mammalian enzyme. *P. denitrificans* is the first system described in which mutagenesis in any complex I core subunit may be combined with quantitative proton-pumping measurements for mechanistic studies.

## Introduction

Mitochondrial complex I, one of the largest and most complicated enzymes in the mammalian cell, is pivotal for energy transduction. It exploits the energy from NADH oxidation by ubiquinone to drive protons across the inner mitochondrial membrane, supporting the proton-motive force that powers ATP synthesis. The 45 subunits of the mammalian complex comprise 14 core subunits, which house the catalytic machinery and are conserved in all species of complex I, and a cohort of 31 supernumerary subunits that is particular to the mammalian complex (1–3). The structures of the core subunits have been described in complex I from several diverse species: a bacterium (*Thermus thermophilus*), a yeast (*Yarrowia lipolytica*), and two mammals (*Bos taurus* and *Ovis aries*) (4–7). These structures have revealed the architecture of the catalytic machinery and provide a foundation for mechanistic studies. In the redox reaction, NADH is oxidized by a flavin mononucleotide at the top of the hydrophilic domain. The electrons then pass down a chain of iron-sulfur clusters to ubiquinone, with its headgroup bound ∼20 Å above the membrane plane. The mechanism by which the redox reactions of the hydrophilic domain initiate a cascade of events leading to proton transfer at distant sites in the membrane domain remains unknown.

A fundamental property of the mechanism of complex I catalysis is the stoichiometry of proton translocation: how many protons does complex I transport across the membrane for each (two-electron) oxidation of NADH? The proton stoichiometry for complex I is considered historically to be four, and so four proton channels have been proposed in structural models (4, 5, 8). However, the locations of the proposed channels vary: there is strong evidence for a tripartite repeat of proton-transporting motifs in three antiporter-like subunits, but the location of the fourth channel is less clear.

In 2005, Hinkle (9) reviewed all the major studies of the ADP phosphorylation/O_2_ reduction (P/O)^2^ ratios of mitochondrial respiration since 1937 and concluded that “values of about 2.5 with NADH-linked substrates and 1.5 with succinate are consistent with most reports.” Hinkle assumed that mammalian F_1_F_0_-ATP synthase requires 9 H^+^ to generate 3 ATP molecules and, by considering the energetic requirements of ADP^3−^/ATP^4−^ exchange and phosphate uptake (10–12), deduced that oxidation of one NADH molecule by complexes I, III, and IV resulted in translocation of 10 H^+^, whereas oxidation of one succinate molecule by complexes II, III, and IV resulted in translocation of 6 H^+^. The latter value is now well established (13–15), indicating that the proton stoichiometry of complex I is four, because complex II does not translocate protons. Work by Wikström (16) using the succinate:O_2_ proton stoichiometry to calibrate the response of pH-sensitive dyes to NADH-linked reactions in intact mitochondria was particularly influential in promoting the same 4 H^+^/2 e^−^ value, supported later by Vinogradov and co-workers (17), who used phenol red to measure the transient pH changes generated by complex I catalysis in submitochondrial particles (SMPs). Recently, however, Wikström and Hummer (45) reappraised both Wikström's and Hinkle's earlier analyses in response to the 8 H^+^/3 ATP stoichiometry of mammalian ATP synthase inferred from the 8 *c*-subunits observed in the crystal structure (18) and concluded that the proton stoichiometry of complex I is three, not four. Subsequently, Ripple and co-workers (19) utilized the *b*-hemes of complex III in intact cells to determine the redox span (Δ*E*) and proton-motive force (Δ*p*) across complex I and then extrapolated to the position of zero net catalysis at which 2Δ*E* = *n*Δ*p*. The data gave a proton stoichiometry (*n* value) close to four. However, in addition to the extensive extrapolation required, the method rests on many assumptions about redox equilibrium between the *b*-hemes and the ubiquinone pool during catalysis (which require accurate knowledge of the *b*-heme potentials in a highly complex environment) and on the accuracy of a stochastic model for complex III catalysis.

Notably, few robust stoichiometry measurements have been made on any complex I other than the bovine enzyme. The proton stoichiometry of *Y. lipolytica* complex I was reported to be 3.8 using the pH-sensitive dye neutral red in intact mitochondria and estimated to be 3–4 using phenol red with complex I reconstituted in proteoliposomes (20). The proton stoichiometry of *Escherichia coli* complex I was found to be “at least” 3 by using a pH electrode to monitor external pH changes upon addition of O_2_ or DMSO to activate complex I catalysis (21). Thus, the possibility that different species of complex I adopt different stoichiometries cannot be excluded: the complex I proton-pumping machinery is modular, marked variations between the core subunits exist between species, and some species use alternative quinones with much lower reduction potentials that imply an altered quantitative scale for bioenergetics. Importantly, these “different species” include the model systems exploited in mechanistic investigations of complex I catalysis, which are assumed to be relevant to the mammalian complex.

Here, we describe a simple and transparent method that uses inverted membrane vesicles to measure the proton stoichiometry of complex I in a bacterial and a mammalian species. Our method relies on the known stoichiometry of 6 H^+^/2 e^−^ for succinate:O_2_ oxidoreduction and assumes that the rate of ATP synthesis depends on Δ*p*. We re-establish the 4 H^+^/2 e^−^ stoichiometry for mammalian complex I and demonstrate, for the first time, the same stoichiometry in a bacterial complex from *Paracoccus denitrificans*. Our results reaffirm the relevance of using simpler model systems for mechanistic studies and enable accurate stoichiometry measurements in a genetically tractable model system for the future testing of mechanistic hypotheses.

## Results

### 

#### 

##### Driving ATP Synthesis by Substrate Oxidation in Coupled Vesicles

**FIGURE 1.**
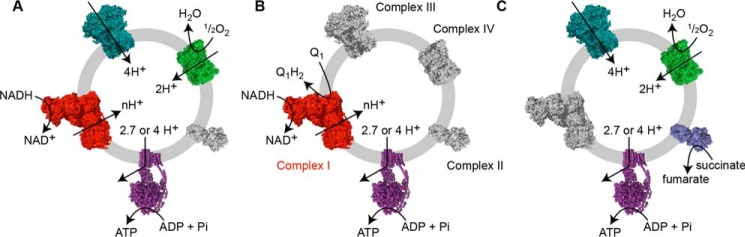
**Schematic representation of ATP synthesis in the SMP and SBP systems.** *A*, the NADH:O_2_ reaction drives proton translocation by complexes I, III, and IV ((*n* + 6) H^+^ per NADH). *B*, the NADH:Q_1_ reaction drives proton translocation by complex I (*n* H^+^ per NADH); complexes III and IV are inhibited. *C*, the succinate:O_2_ reaction drives proton translocation by complexes II, III, and IV (6 H^+^ per succinate). The number of protons required to synthesize 1 ATP is 2.7 in *B. taurus* and 4 in *P. denitrificans*.

We use two model systems: SMPs from bovine heart mitochondria and sub-bacterial particles (SBPs) from *P. denitrificans*. SMPs, formed by sonication of mitochondria to “pinch” off the cristae (22, 23), are sealed membrane vesicles with the matrix side of the membrane (and the NADH-, succinate-, and ATP-binding sites of complexes I and II and ATP synthase, respectively) exposed to the external solution (Fig. 1) to enable direct measurements of substrate oxidation and ATP production. SBPs are topologically equivalent but formed by the osmotic lysis of lysozyme-digested *P. denitrificans* cells (24). In both preparations, the rate of NADH:O_2_ oxidoreduction increases significantly when Δ*p* is dissipated by addition of an uncoupler, showing that they sustain a substantial Δ*p* to drive ATP synthesis. In addition to its homologues of mammalian complexes III and IV, *P. denitrificans* can also express a quinol oxidase (*ba*_3_) and a high O_2_ affinity cytochrome oxidase (*ca*_3_) (15, 25). However, under the aerobic growth conditions used here, neither the high affinity oxidase (26) or the quinol oxidase were expressed to substantial levels; when myxothiazol was used to inhibit complex III, the rate of NADH:O_2_ oxidoreduction was negligible, only ∼2.5% of the inhibitor-free value. The normal *P. denitrificans* electron transport chain also includes two hydrogenases that may oxidize atmospheric H_2_ and reduce quinone; they were deleted from its genome to generate the strain used here (see “Experimental Procedures”).

NADH:O_2_ and succinate:O_2_ oxidoreduction (referred to from hereon as the NADH:O_2_ and succinate:O_2_ reactions) are catalyzed during steady-state respiration by complexes I + III + IV and II + III + IV, respectively (Fig. 1). Complexes I and II oxidize NADH and succinate, respectively, and reduce ubiquinone (typically ubiquinone-10) to ubiquinol. Complex III reoxidizes the ubiquinol and uses cytochrome *c* to pass the electrons to complex IV for the reduction of O_2_ to H_2_O. For each ubiquinol, complexes III and IV transport six protons across the membrane (13–15). Complex II does not transport any protons across the membrane. The number of protons transported for each NADH oxidized by complex I (*n*) is the subject of this study. Thus, the NADH:O_2_ and succinate:O_2_ reactions transport (*n* + 6) and 6 protons, respectively, for each two-electron substrate oxidation cycle, whereas complex I alone transports *n* protons. To measure the complex I only rate, the complex III + IV segment of the chain is inhibited, and ubiquinone-1 (a hydrophilic ubiquinone-10 analogue) is provided to sustain NADH oxidation (the NADH:Q_1_ reaction; Fig. 1*B*). In all three cases, proton transport forms a Δ*p* across the vesicular membrane that is harnessed by ATP synthase to produce ATP from ADP and inorganic phosphate. Here, we use the rate of ATP synthesis as a proxy for the rate of proton translocation by the electron transport chain and compare substrate/ATP ratios for the NADH:O_2_, NADH:Q_1_ and succinate:O_2_ reactions to determine the unknown value of *n* for complex I.

##### Optimizing the Conditions for Measurements

**FIGURE 2.**
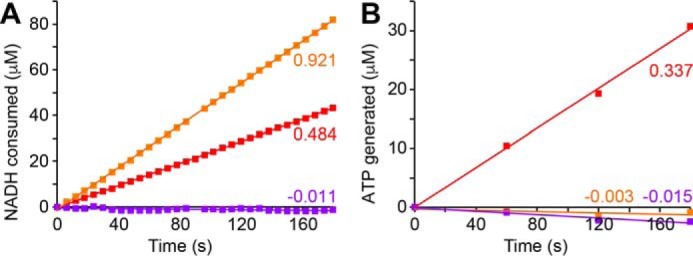
**ATP synthesis driven by the NADH:O_2_ reaction in SMPs is sensitive to dissipation of Δ*p* and inhibition of complex I.** NADH oxidation (*A*) and ATP synthesis (*B*) were initiated with 200 μm NADH and monitored simultaneously (see “Experimental Procedures”). The data from the standard reaction (*red*) are compared with data recorded in the presence of 4 μm FCCP to dissipate Δ*p* (*orange*) and in the presence of 5 μm piericidin A to inhibit complex I catalysis (*magenta*). Rates of NADH oxidation and ATP synthesis are marked in μmol min^−1^ mg^−1^.

Fig. 2 shows data from an experiment in which the NADH:O_2_ reaction was used to drive ATP synthesis in SMPs. NADH oxidation was measured spectroscopically in real time, and ATP synthesis was quantified by removing and testing aliquots of the reaction mixture. To simplify the experiments, a 20-s preincubation with NADH was included, before addition of ATP, to make both rates linear throughout the measurement: complex I catalysis often exhibits a non-linear lag phase caused by slow activation of the “deactive” form of the complex (27), formed when the complex is dormant during its preparation. Activating all the complexes before initiating ATP synthesis also avoids complications from the Na^+^/H^+^ antiporter activity of the deactive enzyme (28). The succinate reaction also exhibited linear catalysis, following a short lag phase from the fumarate (*i.e.* oxidized succinate) detection system, but for the NADH:Q_1_ reaction the insolubility of ubiquinone-1 limited its concentration to 150 μm, and both substrate consumption and product accumulation slowed the reaction at longer assaying times. NADH:Q_1_ reactions were thus limited to 150 s. In addition, piericidin A-insensitive rates of NADH oxidation, which arise from a known side reaction between ubiquinone-1 and the reduced flavin (29), were determined and subtracted.

The addition of piericidin A to inhibit complex I catalysis prevents both NADH oxidation and ATP synthesis, and addition of the protonophore carbonyl cyanide 4-(trifluoromethoxy)phenylhydrazone (FCCP), which dissipates Δ*p* by allowing free proton movement across the membrane, both increases NADH oxidation and prevents ATP synthesis (Fig. 2). Thus, ATP synthesis is driven only by the Δ*p* formed by the respiratory chain. Note that diadenosine pentaphosphate (30) was added to both SMPs and SBPs to inhibit adenylate kinase and prevent it catalyzing Δ*p*-independent ATP formation. In addition, a truncated hexahistidine-tagged form of the bovine ATP synthase inhibitor protein IF_1_ (31) was added to SMPs to inhibit ATP hydrolysis by poorly coupled vesicles, which are known to be present in the heterogeneous SMP preparation (23). IF_1_ inhibits only ATP hydrolysis, not ATP synthesis, so it makes the bidirectional ATP synthase in SMPs behave like the unidirectional *P. denitrificans* ATP synthase in SBPs. The unidirectional behavior of the *P. denitrificans* enzyme has been ascribed to its IF_1_-like ζ-subunit (32, 33). SMP experiments included enough IF_1_ (3.1 μm) to prevent hydrolysis of synthesized ATP and maintain it at a steady concentration for at least 5 min after Δ*p* was dissipated with FCCP.

Finally, in both SMPs and SBPs, a considerable fraction of the protons pumped are lost to leak. Although rates of leak are expected to be relatively low under phosphorylating conditions (34), we set Δ*p* (monitored via the rate of ATP synthesis) to be equal in each of the three reactions we compared by partially inhibiting the two fastest substrate oxidation reactions. This strategy avoids confounding effects from Δ*p*-dependent variations in both the rate of leak (35, 36) and the activity of ATP synthase (37). The inhibitions required were estimated from inhibition *versus* activity curves then fine-tuned by trial and error. For SMPs, the NADH:O_2_ and succinate:O_2_ reactions were inhibited using 13 mm ADP-ribose, a complex I flavin site inhibitor (38), and 5 nm atpenin, a complex II inhibitor (39), respectively. For SBPs, the NADH:O_2_ reaction was inhibited using 8 mm ADP-ribose and the NADH:Q_1_ reaction by 1.25 μm myxathiazol (by increasing the concentration beyond that required to inhibit complex III, we took advantage of its mild inhibition of complex I (40)). The effects of these set inhibitor concentrations on the rates of substrate oxidation are given in Table 1.

**TABLE 1:** **Catalytic rates for the three reactions used for the stoichiometry measurements** In SMPs, the NADH:O_2_ and succinate:O_2_ reactions were inhibited with 13 mm ADP-ribose and 5 nm atpenin, respectively (the NADH:Q_1_ reaction was not inhibited). In SBPs, the NADH:O_2_ reaction was inhibited by 8 mm ADP-ribose, and the NADH:Q_1_ reaction was slowed by 1.25 μm myxathiazol (the succinate:O_2_ reaction was not inhibited). Piericidin A-insensitive rates have been subtracted for the NADH:Q_1_ reaction.

	Rate of reaction		
	NADH:O_2_	Succinate:O_2_	NADH:Q_1_
	μmol min^−1^ mg^−1^		
SMPs not inhibited	0.951 ± 0.008	1.019 ± 0.057	0.703 ± 0.028
SMPs inhibited	0.281 ± 0.010	0.465 ± 0.026	0.703 ± 0.028
SBPs not inhibited	1.262 ± 0.086	0.942 ± 0.039	1.732 ± 0.141
SBP inhibited	0.564 ± 0.011	0.942 ± 0.039	1.393 ± 0.036

##### The Stoichiometries of the Mammalian and Bacterial Complexes I

**FIGURE 3.**
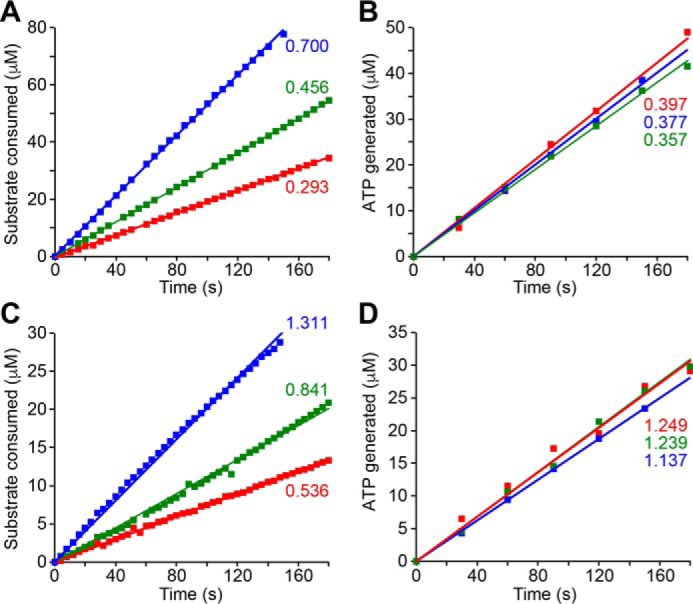
**Data from experiments to determine the stoichiometry of *B. taurus* complex I in SMPs and *P. denitrificans* complex I in SBPs.** *A* and *B* show data from SMPs and *C* and *D* data from SBPs. The rates of substrate consumption (*A* and *C*) and ATP synthesis (*B* and *D*) were monitored simultaneously (see “Experimental Procedures”). Rates of ATP synthesis have been matched using inhibitors (see text). The NADH:O_2_ reaction (*red*) was initiated by the addition of 200 μm NADH in the presence of 13 mm (SMPs) or 5 mm (SBPs) ADP-ribose. The succinate:O_2_ reaction (*green*) was initiated by 5 mm succinate in the presence of 5 nm atpenin (SMPs). The NADH:Q_1_ reaction (*blue*) was initiated by 200 μm NADH in the presence of 150 μm ubiquinone-1 and 500 nm (SMPs) or 1.25 μm (SBPs) myxothiazole. Rates of substrate oxidation and ATP synthesis are marked in μmol min^−1^ mg^−1^; the inhibitor-insensitive rates of the NADH:Q_1_ reaction (0.091 ± 0.011 in SMPs and 0.101 ± 0.009 μmol min^−1^ mg^−1^ in SBPs) have been subtracted.

Fig. 3 shows the substrate oxidation rates and the matched rates of ATP synthesis in experiments on both SMPs and SBPs. The combination of three independent reactions, one with a known H^+^/2 e^−^ stoichiometry and two with unknown stoichiometries, allows three pairwise comparisons to be made, and robust conclusions to be drawn if all three comparisons “triangulate” on a common value.

To make the pairwise comparisons we used Equations 5–7, which are derived as follows.

(i) As described by Mitchell (41), proton fluxes across coupling membranes can be considered as a set of proton circuits. Thus, as an example, Equation 1 describes the relationship between the rate of the succinate:O_2_ reaction (ν_succinate:O_2__) and its associated rate of ATP synthesis (ν_ATP_) during steady-state catalysis. ν_succinate:O_2__ is multiplied by 6 because 6 protons are pumped across the membrane per succinate oxidized, and *p* is the number of pumped protons required to make one ATP molecule (2.7 in mammals or 4.0 in *P. denitrificans*). The third term in Equation 1, ν_Leak_, is the rate at which protons pumped across the membrane by the succinate:O_2_ reaction leak back across, without being used to synthesize ATP. Because the proton fluxes caused by ATP synthesis and leak are in the opposite direction to that from proton pumping, all three terms in Equation 1 are defined as positive quantities.

(Eq. 1) $$6.v_{\text{Succinate}:{\text{O}}_{2}}=p.v_{\text{ATP}}+v_{\text{Leak}}$$

(ii) Combining Equation 1 with an equivalent equation for the NADH:O_2_ reaction (in which the 6 is replaced by (6 + *n*), where *n* is the number of protons pumped by complex I per NADH oxidized) leads to Equation 2. Equation 2 can be derived because the rates of ATP synthesis in the two reactions are equal; thus the Δ*p* values are equal, and so are the ν_Leak_ values.

(Eq. 2) $$6.v_{\text{Succinate}:{\text{O}}_{2}}=\left(6+n\right).v_{\text{NADH}:{\text{O}}_{2}}$$

Using Equation 2 (and equivalent equations) the value of *n* can be calculated for each experimental pairwise comparison. However, Equation 2 only applies in the perfect case, when the two rates of ATP synthesis are truly identical: it cannot account for any experimental error.

(iii) To describe two imperfectly matched rates of ATP synthesis, Equation 1 is first re-written as Equation 3,

(Eq. 3) $$\left(1-k_{\text{Leak}}\right).6.v_{\text{Succinate}:{\text{O}}_{2}}=p.v_{\text{ATP}}$$

where *k*_Leak_ = *v*_Leak_/(6.*v*_Succinate:O_2__). The variable *k*_Leak_ represents the fraction of the pumped protons that are lost to leak (and (1 − *k*_Leak_) is the fraction used to synthesize ATP). For example, if *k*_Leak_ = 0.5, then half the pumped protons are lost, and the effective number needed to synthesize one ATP molecule doubles.

For the example of a comparison between the succinate:O_2_ and NADH:O_2_ reactions, Equation 3 is combined with its equivalent for the NADH:O_2_ reaction, leading to Equation 4 by the elimination of *p*. The subscripts N:O_2_ and S:O_2_ refer to the NADH:O_2_ and succinate:O_2_ reactions, respectively.

(Eq. 4) $$\frac{6+n}{6}=\frac{v_{\text{Succinate}:{\text{O}}_{2}}}{v_{\text{NADH}:{\text{O}}_{2}}}\cdot \frac{v_{\text{ATP}\left(\text{N}:{\text{O}}_{2}\right)}}{v_{\text{ATP}\left(\text{S}:{\text{O}}_{2}\right)}}\cdot \frac{1-k_{\text{Leak}\left(\text{S}:{\text{O}}_{2}\right)}}{1-k_{\text{Leak}\left(\text{N}:{\text{O}}_{2}\right)}}$$

(iv) Equation 4 contains the measured rates of substrate oxidation and ATP synthesis for both reactions. It therefore accounts for imperfect matching in the rates of ATP synthesis (in our experiments pairs of rates varied by 7 ± 4%). However, Equation 4 also contains the unknown ratio between (1 − *k*_Leak(S:O_2_)_) and (1 − *k*_Leak(N:O_2_)_). In a perfect experiment, the rates of proton pumping, ATP synthesis and leak are perfectly matched so (1 − *k*_Leak(S:O_2_)_) = (1 − *k*_Leak(N:O_2_)_), and their ratio is 1. Otherwise, Δ*p*-dependent variations in *k*_Leak(S:O_2_)_ and *k*_Leak(N:O_2_)_ may lead the ratio to deviate from 1. To exclude these variations as a substantial source of error, we used SMPs to test how the ratios of ATP synthesis and substrate oxidation rates depend on inhibitor concentration, at close to the set inhibitor concentrations determined above. Equation 3 shows that (for example) if ν_succinate:O_2__/ν_ATP_ is constant, then *k*_Leak_ is also constant. The data in Table 2 confirm that, for each reaction, the ratios of the rates, and thus the *k*_Leak_ values, do not vary significantly. Consequently, we set the ratios of the (1− *k*_Leak_) values for each pair of reactions to be equal to 1.

**TABLE 2:** **The effects of inhibiting substrate oxidation to different levels on the ratio of ATP production to substrate oxidation** ATP synthesis was carried out using SMPs as described under “Experimental Procedures,” and the substrate oxidation reactions were inhibited as in Table I but using three different inhibitor concentrations for each reaction. The values in bold correspond to the inhibitor concentrations used in Table I and for stoichiometry determination.

	NADH:O_2_			Succinate:O_2_			NADH:Q_1_		
Inhibition of substrate oxidation (%)	61.3 ± 4.9	**69.7 ± 1.6**	84.0 ± 1.3	38.2 ± 1.8	**52.7 ± 2.6**	67.1 ± 0.3	**0.0 ± 6.0**	13.7 ± 2.7	30.4 ± 0.6
ATP/2 e^−^	1.29 ± 0.19	**1.35 ± 0.08**	1.35 ± 0.11	0.79 ± 0.04	**0.81 ± 0.05**	0.83 ± 0.01	**0.54 ± 0.04**	0.53 ± 0.02	0.55 ± 0.01

(v) Following the derivations in (i)–(iv), Equations 5–7 were used for the three pairwise comparisons, where the N:Q_1_ subscript is used to denote the NADH:Q_1_ reaction.

(Eq. 5) $$\frac{6+n}{6}=\frac{v_{\text{Succinate}:{\text{O}}_{2}}}{v_{\text{NADH}:{\text{O}}_{2}}}\cdot \frac{v_{\text{ATP}\left(\text{N}:{\text{O}}_{2}\right)}}{v_{\text{ATP}\left(\text{S}:{\text{O}}_{2}\right)}}$$

(Eq. 6) $$\frac{n}{6}=\frac{v_{\text{Succinate}:{\text{O}}_{2}}}{v_{\text{NADH}:{\text{Q}}_{1}}}\cdot \frac{v_{\text{ATP}\left(\text{N}:{\text{Q}}_{1}\right)}}{v_{\text{ATP}\left(\text{S}:{\text{O}}_{2}\right)}}$$

(Eq. 7) $$\frac{6+n}{n}=\frac{v_{\text{NADH}:{\text{Q}}_{1}}}{v_{\text{NADH}:{\text{O}}_{2}}}\cdot \frac{v_{\text{ATP}\left(\text{N}:{\text{O}}_{2}\right)}}{v_{\text{ATP}\left(\text{N}:{\text{Q}}_{1}\right)}}$$

Tables 3 and 4 present the results from combining the data from four independent experiments on each system with Equations 5–7 to give the stoichiometry of complex I in each system. The values derived from the data in Fig. 3 are given in the first rows of the tables. Table 3 strongly supports 4 H^+^/2 e^−^ as the proton pumping stoichiometry of mammalian complex I, in line with a subset of previous measurements (16, 17). Table 4 presents the first accurate measurement of the H^+^/2 e^−^ stoichiometry of complex I in a bacterial system, *P. denitrificans*; the values determined also support 4 H^+^/2 e^−^, indicating that the proton stoichiometry is conserved in different species of complex I.

**TABLE 3:** **Complex I proton pumping stoichiometry measurements in SMPs** The results from four independent complex I stoichiometry measurements in *B. taurus* SMPs, each with errors propagated from the standard errors in the rate measurements, are given. In the last column the efficiency (which is equal to (1 − *k*_Leak_) × 100%) is reported as the average of the values from the three reactions. The bottom row displays the averages from all four measurements, with propagated errors.

	Stoichiometry from comparison of pairs of reactions			Efficiency of ATP synthesis assuming 4 H^+^/2 e^−^ for complex I
	NADH:O_2_ *vs*. succinate:O_2_	NADH:Q_1_ *vs*. succinate:O_2_	NADH:O_2_ *vs*. NADH:Q_1_	
Experiment 1	4.39 ± 0.09	4.13 ± 0.07	3.95 ± 0.08	35.6 ± 0.4
Experiment 2	3.78 ± 0.11	3.63 ± 0.08	3.54 ± 0.09	42.5 ± 1.2
Experiment 3	4.24 ± 0.16	4.23 ± 0.12	4.23 ± 0.16	38.3 ± 0.6
Experiment 4	4.42 ± 0.06	4.41 ± 0.16	4.40 ± 0.17	40.3 ± 1.1
Average	4.21 ± 0.15	4.10 ± 0.17	4.02 ± 0.19	39.2 ± 3.0

**TABLE 4:** **Complex I proton pumping stoichiometry measurements in SBPs** The results from four independent complex I stoichiometry measurements in *P. denitrificans* SBPs each with errors propagated from the standard errors in the rate measurements, are given. In the last column the efficiency (which is equal to (1 − *k*_Leak_) × 100%) is reported as the average of the values from the three reactions. The bottom row displays the averages of all four measurements, with propagated errors.

	Stoichiometry from comparison of pairs of reactions			Efficiency of ATP synthesis assuming 4 H^+^/2 e^−^ for complex I
	NADH:O_2_ *vs*. succinate:O_2_	NADH:Q_1_ *vs*. succinate:O_2_	NADH:O_2_ *vs*. NADH:Q_1_	
Experiment 1	3.72 ± 0.24	3.86 ± 0.14	3.96 ± 0.24	59.4 ± 0.6
Experiment 2	3.50 ± 0.11	3.53 ± 0.06	3.55 ± 0.11	92.7 ± 3.3
Experiment 3	4.57 ± 0.22	4.38 ± 0.19	4.25 ± 0.17	78.5 ± 2.1
Experiment 4	4.24 ± 0.18	4.13 ± 0.18	4.06 ± 0.22	89 ± 0.9
Average	4.01 ± 0.24	3.98 ± 0.18	3.96 ± 0.15	80.1 ± 14.0

##### Efficiency of ATP Synthesis in Coupled Vesicles

By using the known *c*-ring stoichiometries of the ATP synthases (18, 33) to define the H^+^/ATP stoichiometries as 8/3 and 12/3 for *B. taurus* and *P. denitrificans*, respectively, together with the stoichiometry of complex I determined above, measured substrate/ATP ratios can be used to calculate *k*_Leak_ values from equation 3 (and equivalent equations for the other substrate reactions). For convenience we then define the “efficiency of ATP synthesis” as (1 − *k*_Leak_) × 100%. Table 3 shows that the efficiency of ATP synthesis by SMPs is 39 ± 3%. The efficiency is the same for all three reactions (individual values can be calculated using the data in Fig. 3), confirming that the *k*_Leak_ values for each reaction are also the same. The efficiency of ATP synthesis by SBPs is markedly higher: 80 ± 14% for all three reactions (Table 4). The difference may be caused by higher proton leak in SMPs, by natural differences in membrane composition or morphology, or by a different “quality” of vesicle formation. Notably, in the absence of inhibitors, efficiencies of ATP synthesis are lower (the steady-state Δ*p* values are higher, and so leak is greater) and vary between the reactions. For example, for uninhibited NADH:O_2_-driven ATP synthesis in SMPs (Fig. 2), the efficiency is only 20 ± 2%. This result underlines the need to approximately match the rates of ATP synthesis in pairwise comparisons. Indeed, a trend exists between efficiency and respiratory control ratio (RCR), the ratio of the rates of catalysis in the absence and presence of Δ*p*, which is often taken as a measure of how well the vesicles are sealed. For the NADH:O_2_ reaction our SBPs have an RCR of 4.03 ± 0.06, compared with 3.20 ± 0.29 for the SMPs. Although it is tempting to extrapolate this trend to intact mitochondria, for example the ∼90% efficiency that has been reported for rat liver mitochondria with RCR values of ∼10 respiring on glutamate and malate (42), substrate oxidation by intact mitochondria requires additional substrate transport and conversion processes and is not directly comparable.

Finally, we attempted to use our method to determine the H^+^/2 e^−^ stoichiometry for complex I in SBPs prepared from *E. coli*. First, because *E. coli* expresses several alternative NADH: quinone oxidoreductases (single subunit enzymes that do not transport protons), experiments were carried out using deamino-NADH, a substrate considered selective for complex I that provides a simple way to circumvent this issue (43). Deamino-NADH oxidation by the *E. coli* SBPs was confirmed to be fully sensistive to the inhibitor piericidin A. Second, because of the insensitivity of the *E. coli* ubiquinol oxidases to standard inhibitors, the NADH:Q_1_ reaction was carried out anaerobically. The rate of the NADH:O_2_ reaction was similar to in SMPs and SBPs (0.459 ± 0.063 μmol min^−1^ mg^−1^), but within individual experiments the three ratios determined did not triangulate, and repeated experiments did not reach any consensus on the stoichiometry. It is possible that the low rates of ATP synthesis observed (0.113 ± 0.05 μmol min^−1^ mg^−1^ for the NADH:O_2_ reaction) are an issue, leading to unacceptable levels of uncertainty and irreproducibility. In turn, the low ATP synthesis may result from high proton leak across the vesicular membrane (either an intrinsic property of the *E. coli* membrane composition, or a result of the vesicle preparation) and/or from the lower proton-pumping stoichiometries of the three reactions. In place of complexes III and IV, the *E. coli* strain used expresses the cytochrome *bo*_3_ (2 H^+^/e^−^) quinol oxidase (cytochrome *bd*-I is not expressed under the aerobic growth conditions used, and cytochrome *bd*-II has been genetically ablated). Therefore the *E. coli* proton stoichiometries are (*n* + 4) for the NADH:O_2_ reaction, 4 for the succinate:O_2_ reaction, and *n* for the NADH:Q_1_ reaction. Consistent with all these suggestions, the RCR values of our *E. coli* vesicles were low (∼1.2, typical of values from the literature (44)), and efficiencies of ATP synthesis were also low: 11 ± 6% for the NADH:O_2_ reaction (assuming the ATP synthase *c*-ring stoichiometry is 10).

## Discussion

Our results confirm that the proton-pumping stoichiometry of mammalian complex I is 4 H^+^/2 e^−^, consistent with the consensus of earlier measurements (16, 17, 19), not 3 H^+^/2 e^−^ as proposed recently by Wikström and Hummer (45). Furthermore, we demonstrate, for the first time, that complex I from a bacterial species, *P. denitrificans*, also has a stoichiometry of 4 H^+^/2 e^−^.

Wikström and Hummer based their 3 H^+^/2 e^−^ stoichiometry for complex I on two arguments (45). First, for catalysis to occur, the Gibbs free energy available from the redox reaction (−2Δ*E*) must be greater than that required for proton translocation (*n*Δ*p*). With the values Wikström and Hummer chose for Δ*E* (∼0.359 V) and Δ*p* (0.22–0.23 V), this is only true if *n* = 3, not if *n* = 4. However, to determine Δ*E* and Δ*p* accurately requires technically challenging measurements of Δ*p* and of the NAD^+^/NADH and ubiquinone/ubiquinol ratios, together with accurate knowledge of the reduction potentials under the specific conditions present—all in aerobically respiring mitochondria. Furthermore, the values used for the comparison were drawn from different sources. Thus, the discrepancy likely arose from inaccuracy or incompatibility in the values used. Second, Wikström and Hummer selected the results of a single study (46) for their evaluation of P/O ratios from intact mitochondria. These results lead to a complex I proton stoichiometry of 2.82 ± 0.29 (Table 5). In contrast, considering all the values collated by Hinkle in his meta-analysis yields 3.57 ± 1.63 and using all the values reported since 1975 (to exclude early values obtained using manometers) leads to 4.20 ± 1.31 (Table 5). Because of the large range in experimental data, these more comprehensive analyses do not ambiguously exclude a value of 3, but they clearly center on 4, matching the value determined here.

**TABLE 5:** **Calculations of the stoichiometry of complex I using published P/O ratios** The stoichiometry of complex I was calculated using the single data set chosen by Wikström and Hummer (45, 46), using all of the values collated by Hinkle in 2005 (9), and using all of the values reported from 1975 onwards collated by Hinkle. For the single study (46), the errors are the experimental errors reported from that study. For the values collated by Hinkle, the standard deviations describe the variation between studies. The final stoichiometry values have been calculated using 1.5 for the known P/O ratio of complexes III + IV.

Reactions considered	Complexes that contribute to Δ*p*	Single data set	All studies	Studies from 1975
**P/O ratios**				
Reaction A	I + III + IV	2.27 ± 0.08	2.47 ± 0.45	2.64 ± 0.36
Reaction B	III + IV	1.48 ± 0.04	1.56 ± 0.17	1.56 ± 0.17
Reaction C	III	0.49 ± 0.02	0.58 ± 0.21	0.48 ± 0.03
Reaction D	IV	0.98 ± 0.09	1.03 ± 0.15	1.09 ± 0.18
A − B	I	0.79 ± 0.09	0.92 ± 0.48	1.08 ± 0.39
A − (C + D)	I	0.80 ± 0.12	0.86 ± 0.51	1.07 ± 0.40

**Complex I H^+^/2 e^−^stoichiometries**				
A − B	I	2.90 ± 0.33	3.36 ± 1.75	3.97 ± 1.45
A − (C + D)	I	2.93 ± 0.45	3.15 ± 1.86	3.93 ± 1.47
A − 1.5	I	2.82 ± 0.29	3.57 ± 1.63	4.20 ± 1.31

Observation of the same stoichiometry value for both a mammalian and a bacterial complex I suggests that it is widely conserved and not affected by the truncation of subunit ND2 in metazoans (47) or by the many supernumerary subunits of the eukaryotic complexes. However, among bacterial complexes I, the *P. denitrificans* enzyme is relatively similar to the mammalian one: *P. denitrificans* is an α-proteobacterium closely related to the protomitochondrion (25), and it uses ubiquinone as its electron acceptor. The *P. denitrificans* complex I subunits share greater identity with their mammalian counterparts than those of *E. coli* or *T. thermophilus*. Here, our attempts to measure the stoichiometry of *E. coli* complex I were unsuccessful; the only published study reported at least three (21), and additional questions on whether it can pump sodium ions instead of protons, perhaps contingent on whether ubiquinone or menaquinone is the substrate (48), remain unresolved. In contrast, complex I in *T. thermophilus* only uses menaquinone; Δ*E* is lower so the enzyme must operate under lower Δ*p* if it is to adopt the same stoichiometry as the mammalian enzyme. These observations suggest caution in extending the common stoichiometry value to all bacterial species and in using uncharacterized bacterial model systems to address the mechanism of the mammalian complex.

Finally, knowing the four-proton stoichiometry of complex I is essential for mechanistic studies. Three antiporter-like subunits, conserved in all the structures determined so far, contain structural hallmarks that indicate their ability to transport protons across the membrane during catalysis, including discontinuous transmembrane helices and conserved charges in the central membrane plane (4–7). Three equivalent pathways and four protons suggest each antiporter-like subunit transports one proton per cycle and that a fourth pathway is also present. The location of the fourth pathway has been proposed to be in subunits ND1, ND4L, and ND6 (4) but is still debated (5). Together, detailed structural information, an accurate method for measuring the proton stoichiometry, and a bacterial model system amenable to site-directed mutagenesis now provide a powerful route to elucidating the mechanism of redox-coupled proton transport in this challenging respiratory enzyme.

## Experimental Procedures

### 

#### 

##### Preparation of Inverted Membrane Vesicles

SMPs from *B. taurus* heart mitochondria were prepared as described previously (23) and SBPs from *P. denitrificans* using a method based on that of John and Whatley (24). *P. denitrificans* cells (strain ΔHy Pd1222, see below) were grown aerobically in two 500-ml cultures in LB medium (30 °C, 225 rpm) and harvested at mid-exponential phase (*A*_600_ = ∼2) by centrifugation (14,000 × *g*, 10 min). The cells were resuspended in 2 liters of 150 mm NaCl, 10 mm Tris-SO_4_ (pH 7.4), and centrifuged again. All the following steps were at 4 °C. The cells were resuspended to an *A*_600_ = ∼7.5 in 10 mm Tris-SO_4_ (pH 7.4), 500 mm sucrose, and 250 μg ml^−1^ egg white lysozyme (Sigma) and incubated for 1 h, and then the digested cells were collected by centrifugation as before. Then they were suspended in 450 ml of 10 mm Tris-SO_4_ (pH 7.4) to drive lysis and vesicle formation. Next, MgSO_4_ (5 mm) and a few flakes of bovine pancreatic DNase (Sigma) were added, and the lysate was centrifuged twice (14,000 × *g*, 15 min), taking the supernatant each time. The final supernatant was centrifuged at 14,000 × *g* for 1 h, and then the pellet containing the SBPs was resuspended in 5 mm Tris-SO_4_ (pH 7.4) and 250 mm sucrose to ∼8 mg ml^−1^. The typical yield was ∼12 mg of SBPs.

The method used to prepare *E. coli* vesicles was modified from that of Burstein *et al.* (44). *E. coli* Keio knock-out strain JW0961 (GE Dharmacon) (lacking *appB* for cytochrome *bd*-II subunit II) was used to inoculate 50 ml of LB medium containing 50 μg ml^−1^ kanamycin and incubated overnight (37 °C, 225 rpm). Then the culture was used to inoculate 0.5 liter of medium in a 2-liter flask to an *A*_600_ of 0.001, and the cells were grown until the mid-exponential phase (*A*_600_ = ∼2). The following steps were performed at 4 °C: the cells were harvested by centrifugation (5,000 × *g*, 15 min) and then resuspended to 10% (w/v) in 10 mm Tris-SO_4_ (pH 7.4), 1 mm EDTA, 1 mm DTT, and 10% (v/v) glycerol. 10 mm MgSO_4_ was added, and the cells were broken by three passages through a cooled Stansted pressure cell homogenizer at 8,000 p.s.i. The cell debris was removed by centrifugation (5,000 × *g*, 10 min), and then the vesicles were collected (175,000 × *g*, 120 min) and resuspended in 10 mm Tris-SO_4_ (pH 7.4 at 32 °C), 5 mm MgSO_4_, and 10% (v/v) glycerol.

##### Generation of the ΔHy Strain of P. denitrificans

**FIGURE 4.**
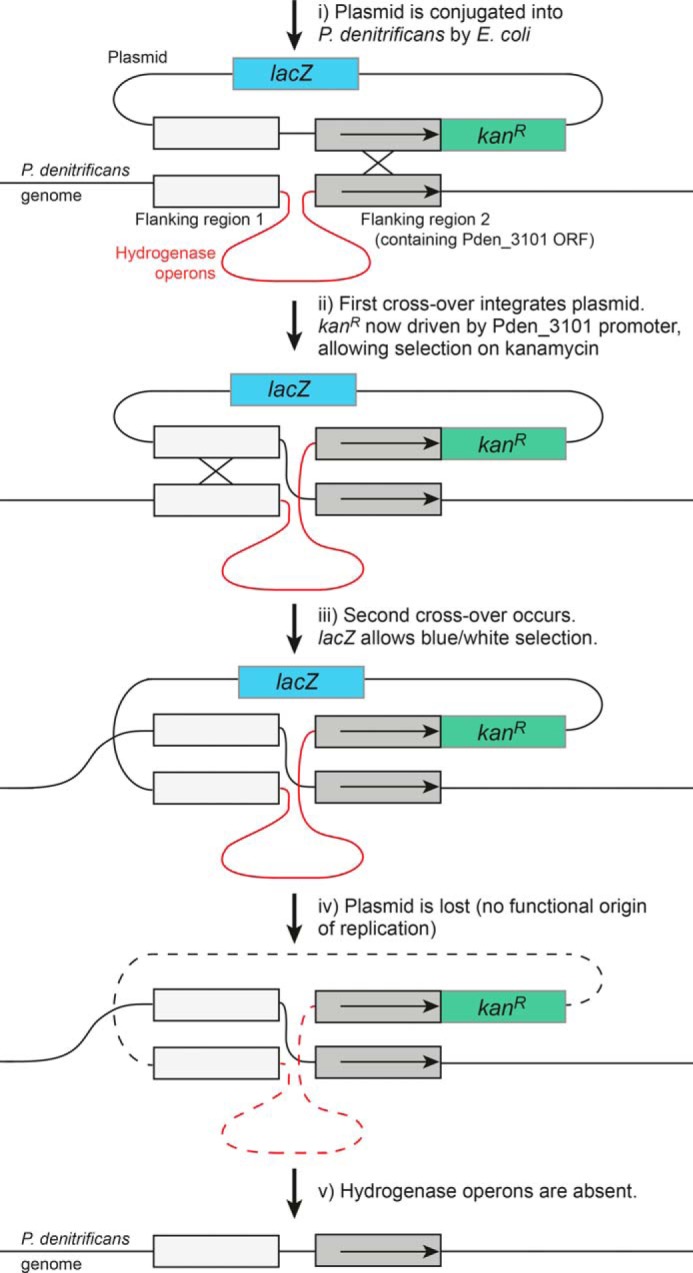
**The strategy taken to delete the two hydrogenase genes in *P. denitrificans*.** *Horizontal arrows* in flanking region 2 show the Pden_3101 ORF and promoter region.

Homologous recombination (Fig. 4) was used to create an unmarked deletion of the two hydrogenase operons (49) identified in the genome of *P. denitrificans* Pd1222 (ORFs Pden_3093 to Pden_3100). The deletion cassette, containing two sequences homologous to regions on each side of the hydrogenase operons followed by *kan*^R^, was assembled by Gibson assembly (50) and placed in the *Eco*R1 site of the *lacZ*-containing pRVS1 plasmid (51). The plasmid was conjugated into *P. denitrificans* (51) using the MFD*pir* strain of *E. coli* to avoid mobilizing *E. coli* genes (52) and plated onto kanamycin (100 μg ml^−1^) to select for colonies that had undergone the first recombination event. Positive colonies were plated onto X-gal (200 μg ml^−1^) and white colonies, which have undergone the second recombination event, selected. Elimination of *kan*^R^ and the plasmid, as well as the hydrogenase operons (nucleotides 276953–287430 of chromosome 2), was confirmed by sequencing and sensitivity to kanamycin.

##### Coupled ATP Synthesis Measurements

All measurements were carried out at 32 °C in 10 mm Tris-SO_4_ (pH 7.5), 250 mm sucrose, 2 mm MgSO_4_, and 25 μm (for SMPs) or 250 μm (for SBPs) diadenosine pentaphosphate. Catalase (*Corynebacterium glutamicum*, 5000 units ml^−1^) and superoxide dismutase (bovine erythrocytes, 20 units ml^−1^) (Sigma) were added to minimize oxidative damage. IF_1_, a truncated hexahistidine-tagged form of the bovine ATP synthase inhibitor protein (comprising residues 1–60) prepared as described previously (31), was added to SMPs at 3.1 μm. NADH:O_2_ oxidoreduction was carried out using 200 μm NADH with its rate modulated by 13 mm (SMPs) or 8 mm (SBPs) ADP-ribose (38); 5 mm succinate was present to minimize differences in solution composition between the different assays, with complex II inhibited fully by 1 μm atpenin (39). NADH:Q_1_ oxidoreduction was carried out in 200 μm NADH, 150 μm ubiquinone-1, 5 mm succinate, and 1 μm atpenin, with 400 μm KCN to inhibit complex IV, and myxothiazol (0.5 μm for SMPs or 1.25 μm for SBPs) to inhibit complex III. KCN was required because Q_1_H_2_ is able to reduce cytochrome *c* directly, bypassing complex III and inducing complex IV catalysis (53). Succinate:O_2_ oxidoreduction was carried out in 5 mm succinate and modulated by 5 nm atpenin in SMPs; succinate oxidation was monitored using a coupled assay system (54). In all cases substrate oxidation was initiated by the addition of 30–40 μg ml^−1^ SMPs, or 8–12 μg ml^−1^ SBPs; then, after 20 s, ATP synthesis was initiated by the addition of 1 mm ADP and 10 mm KPO_4_. NADH and succinate oxidation (followed as NADPH reduction) were monitored spectroscopically at 340–380 nm (ϵ = 4.81 mm^−1^ cm^−1^) in the cuvette housing of a SpectraMax Plus 384 (Molecular Devices) plate reader. ATP synthesis was monitored by withdrawing and quenching aliquots of the reaction mixture, starting immediately and then at 30-s intervals. To quench the reaction, 10 μl of reaction mixture were added to 40 μl of 4% trifluoroacetic acid, and then (after 20 s) 950 μl of 1 m Tris-SO_4_ (pH 8.1) were added to neutralize the pH. ATP concentrations were determined using the Roche ATP Bioluminescence assay kit CLS II in an Autolumat tube luminometer (Berthold), by comparison with known standards.

## Author Contributions

A. J. Y. J. designed the project with J. H., prepared SMPs, and designed and conducted experiments. J. N. B. optimized SBP preparation and carried out *P. denitrificans* genetic manipulations. F. V. prepared SBPs from *P. denitrificans* and membrane vesicles from *E. coli*. J. H. designed the project and experiments with A. J. Y. J. and managed the project. A. J. Y. J. and J. H. wrote the paper with help from the other authors.

## Footnotes

2 The abbreviations used are:

P/O: phosphate (ATP formation) to oxygen (^1^/_2_ O_2_ consumption) ratio
FCCP: 4-(trifluoromethoxy)phenylhydrazone
Q_1_: ubiquinone-1
RCR: respiratory control ratio
SBP: sub-bacterial particle
SMP: submitochondrial particle.
